# Overexpression of *OsC3H10*, a CCCH-Zinc Finger, Improves Drought Tolerance in Rice by Regulating Stress-Related Genes

**DOI:** 10.3390/plants9101298

**Published:** 2020-10-01

**Authors:** So Yoon Seong, Jae Sung Shim, Seung Woon Bang, Ju-Kon Kim

**Affiliations:** 1 Crop Biotechnology Institute, GreenBio Science and Technology, Seoul National University, Pyeongchang 25354, Korea; syseong7@snu.ac.kr (S.Y.S.); jsshim@jnu.ac.kr (J.S.S.); tmddns0903@snu.ac.kr (S.W.B.); 2 Present address: School of Biological Sciences and Technology, Chonnam National University, Gwangju 61186, Korea

**Keywords:** CCCH zinc finger, Rice, Drought tolerance, Processing bodies (PB), Stress granules (SG)

## Abstract

CCCH zinc finger proteins are members of the zinc finger protein family, and are known to participate in the regulation of development and stress responses via the posttranscriptional regulation of messenger RNA in animals and yeast. However, the molecular mechanism of CCCHZF-mediated drought tolerance is not well understood. We analyzed the functions of *OsC3H10*, a member of the rice CCCHZF family. *OsC3H10* is predominantly expressed in seeds, and its expression levels rapidly declined during seed imbibition. The expression of *OsC3H10* was induced by drought, high salinity and abscisic acid (ABA). Subcellular localization analysis revealed that OsC3H10 localized not only in the nucleus but also to the processing bodies and stress granules upon stress treatment. Root-specific overexpression of *OsC3H10* was insufficient to induce drought tolerance, while the overexpression of *OsC3H10* throughout the entire plant enhanced the drought tolerance of rice plants. Transcriptome analysis revealed that *OsC3H10* overexpression elevated the expression levels of genes involved in stress responses, including LATE EMBRYOGENESIS ABUNDANT PROTEINs (LEAs), PATHOGENESIS RELATED GENEs (PRs) and GERMIN-LIKE PROTEINs (GLPs). Our results demonstrated that *OsC3H10* is involved in the regulation of the drought tolerance pathway by modulating the expression of stress-related genes.

## 1. Introduction

Explosive increases in the world population and global climate change have led to a desperate need to use water-deficient areas for crop production. This has necessarily motivated efforts to improve crop productivity under drought conditions. One strategy for improving crop productivity under drought conditions is to develop new crop varieties through the manipulation of drought tolerance mechanisms. Achieving this goal requires a precise understanding of the drought tolerance mechanisms in plants. Plants have evolved numerous adaptive strategies, including developmental and physiological changes, to acclimate to drought stress, many of which are the results of transcriptional reprogramming by drought-induced genes. The drought-induced regulation of the transcriptional network includes transcriptional and posttranscriptional regulation of mRNAs [1,2,3]. It has been reported that genes involved in RNA metabolism are actively involved in drought responses [4,5].

Cysteine3Histidine (CCCH) zinc finger proteins are characterized based on the structure of the zinc-binding motif, which consists of three cysteines and one histidine. CCCH zinc finger proteins (TZFs) are widely conserved in yeast, animals and plants. Genome-wide analysis has revealed 68 and 67 CCCH zinc finger genes in Arabidopsis and rice, respectively [6]. Plant CCCH zinc finger proteins are classified into different subgroups based on the number and pattern of the CCCH motifs. The majority of CCCH zinc finger proteins contain one or two CCCH motifs. Among them, genes containing plant-unique tandem CCCH zinc finger (TZF) motifs preceded by an arginine rich (RR) motif are classified into an RR-TZF subgroup [7]. Plant RR-TZF proteins are involved in the regulation of hormone-mediated growth and stress responses [7,8,9,10]. In Arabidopsis, *AtTZF1,4,5* and *6* regulate seed germination by acting as both positive regulators of abscisic acid(ABA) and negative regulators of gibberellic acid(GA) synthesis [8,9]. *AtTZF1* mediates ABA-dependent abiotic stress tolerance [8]. The overexpression of *AtTZF2* and *AtTZ*F*3* also enhances the tolerance to abiotic stresses [11,12,13]. Additionally, RR-TZF is also known to be involved in biotic stresses. For example, AtTZF9 is phosphorylated by mitogen-activated protein kinases (MAPKs) and is required for pathogen-associated molecular pattern (PAMP)-triggered immune responses [14]. In rice, *OsTZF1/OsC3H35* acts as a negative regulator of several developmental processes, such as seed germination, vegetative growth, and leaf senescence, but also provides tolerance to high-salinity and drought stresses [10].

Several members of the TZF family are implicated in the posttranscriptional regulation of mRNA [15,16]. For example, tristetraproline (TTP), the prototype of mammalian TZF proteins, inhibits tumor necrosis factor-alpha production by destabilizing its mRNA [17]. Yeast Cth2 binds to adenylate-uridylate-rich elements (AU-rich elements) within the 3-UTR of many mRNAs involved in iron utilization to promote their turnover [15]. Similarly, AtTZF1, AtTZF9 and OsTZF1 have been shown to be associated with RNA in vitro as a shuttle between the nucleus and the foci [10,14,18]. Interestingly, AtTZF2 and AtTZF3 possess RNase activity and are able to degrade RNA in vitro [11]. TZF proteins are also proposed as critical components in the nucleocytoplasmic shuttling of mRNA that can localize to processing bodies (PBs) and stress granules (SGs). PBs and SGs are cytoplasmic aggregates formed by the messenger ribonucleoprotein complex which play important roles in posttranscriptional regulation [16,19]. PBs are enriched with translationally inactive mRNAs and degradation machinery, while SGs are the place where translationally fused mRNAs and preinitiation factors are stored [20,21]. In plants, Arabidopsis AtTZF1,4,5,6, and 9 and rice OsTZF1 are reported to be localized in PBs and SGs [9,10,14,18]. Among them, *AtTZF1* and *AtTZF9* are experimentally proven to function in shuttling between the nucleus and the cytoplasm [14,18]. The association of TZF proteins with PBs and SGS is regulated by developmental- and stress-driven signals. The localization of AtTZF1 into cytoplasmic foci is predominantly detected in stomata and meristems under normal conditions. However, the association of AtTZF1 with cytoplasmic foci is detected in other tissues after methyl jasmonate treatments [18]. Similarly, OsTZF1 is mainly localized in the cytoplasm under normal conditions but is localized in PBs and SGs after ABA and salt treatment in rice [10]. Here, we identified rice *OsC3H10*, whose expression is induced by drought and other abiotic stresses, and investigated its putative role in drought tolerance by evaluating the performance of transgenic rice plants with increased expression of *OsC3H10* either throughout the entire plant or specifically in the roots. We also characterized the expression patterns and subcellular localization of *OsC3H10* and identified genes that are related to the *OsC3H10*-mediated drought tolerance pathway.

## 2. Results

### 2.1. OsC3H10 Expression is Induced by Drought

**Figure 1:**
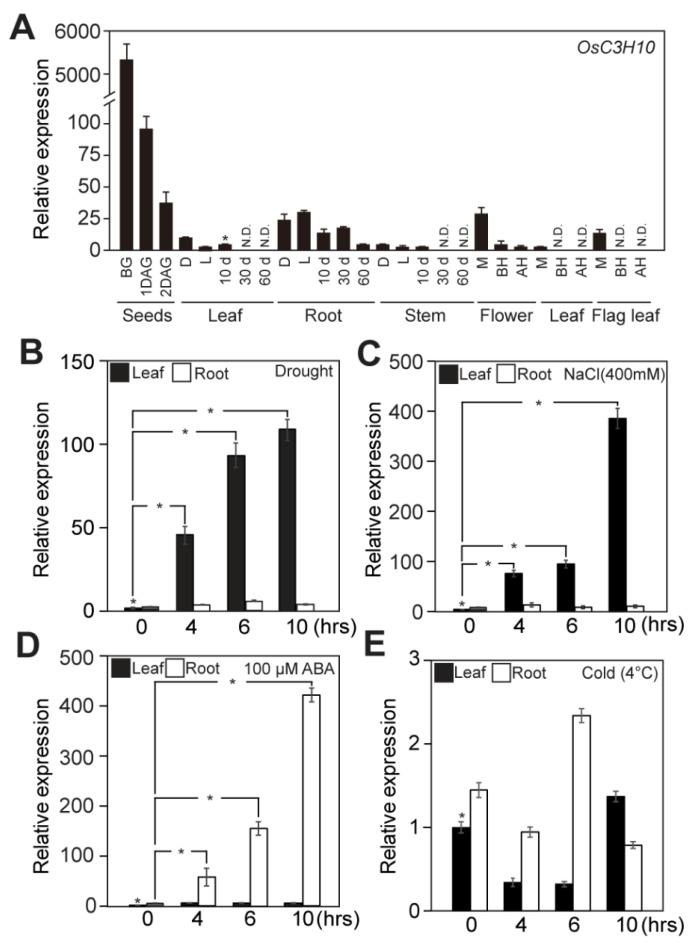
Expression patterns of *OsC3H10* in response to abiotic stress. (**A**) Expression patterns of *OsC3H10* in rice tissues at different developmental stages (*Oryza sativa*. L. Japonica cv. Ilmi). BG, Before Germination; DAG, Day After Germination; d, day; D, Dark; L, Light; M, Meiosis; BH, Before heading; AH, After heading; N.D., Not Determined. (**B–E**) The relative expression patterns of *OsC3H10* in response to four different abiotic stresses. Two-week-old rice seedlings (*Oryza sativa*. L. Japonica cv. Ilmi) were exposed to air-drying (drought) (**B**), 400 mM NaCl (high salinity) (**C**), 100 μM abscisic acid (ABA) (**D**), and 4 °C (**E**) (low temperature) conditions. Leaves and roots of rice plants were harvested at the indicated time points after treatment for gene expression analysis. Rice *UBIQUITIN1* (*OsUBI1*) was used as an internal control for normalization, and 10-d-old leaves were used as positive controls (marked by asterisks). Data represent the mean value ± standard deviation (SD) (*n* = 3). Significant differences from the nontreated control are indicated by asterisks (Student’s *t*-test, * *p* < 0.05).

To identify the rice CCCHs involved in drought tolerance responses, we first examined the expression patterns of *OsCCCH*s using our previously reported RNA sequencing data generated from rice plants exposed to drought stress [22]. In our previous study, non-transgenic plants were exposed to drought for 3 days and leaves of each 0, 1, 2, 3 days after drought were analyzed with RNA sequencing. From this data, we found that the expression of *OsTZF1*, *OsC3H10, 37* and *50* was induced by drought stress in rice plants (Table S1). Such drought-induced expression of those *OsTZF*s was validated by independent qRT-PCR analysis of the rice leaves and roots that were exposed to drought conditions (Figure 1B, Figure S1A). The expression of *OsTZF1*, *OsC3H10* and *37* was induced in leaves under drought conditions, while the expression of *OsC3H50* was upregulated in roots. Among the four drought-induced *OsCCCH* genes, *OsC3H10* was chosen for further study based on its robust expression patterns under drought conditions. The basal expression of *OsC3H10* was higher in roots than in leaves, and showed increase patterns in both leaves and root under drought stress, with higher increase in leaves. (Figure 1B). *OsC3H10* expression was also induced at higher levels in leaves than in roots under high salinity conditions (Figure 1C). Interestingly, the ABA treatment increased the expression of *OsC3H10* in roots more than in leaves (Figure 1D). Similarly, the expression of *OsTZF1*, *OsC3H37*, and *OsC3H50* was predominantly induced in roots by the ABA treatment (Figure S1B). Unlike the other tested abiotic stresses, no significant induction of *OsC3H10* was detected under the low-temperature treatment (Figure 1E). To understand how the *OsC3H10* expression patterns were differently regulated by drought and ABA, we examined the promoter sequence of OsC3H10. ABA-independent drought regulatory genes typically contain a specific *cis*-element known as the dehydration-responsive element/C-repeat (DRE/CRT), A/GCCGAC [23]. DREs or ABA-responsive (ABRE) *cis*-elements were overrepresented in the promoters of *OsTZFs* whose expression was induced by drought treatments (Figure S1C). Specifically, *OsTZF1* contained seven ABREs but no DRE, while the promoters of *OsTZF1, OsC3H10* and *37* included both DREs and ABREs (Figure S1C). These data support the idea that the drought-induced expression of *OsC3H10* is achieved through an ABA-independent pathway. We next determined the temporal expression patterns of *OsC3H10* in various developmental stages of the seeds, leaves, roots, stems, flowers, and flag leaves (Figure 1A). *OsC3H10* transcripts were detected in most tested tissues except in the later stages of leaves, stems, and flag leaves. The expression levels of *OsC3H10* were most abundant in dry seeds and decreased continuously during germination, yet had no increase in germination level (Figure S2). *OsC3H10* expression was generally higher in the early developmental stages of the tested tissues. These data indicate that *OsC3H10* is highly expressed in dry seeds and that its expression is induced by abiotic stresses, such as drought, high salinity and ABA.

### 2.2. Subcellular Localization of OsC3H10

**Figure 2:**
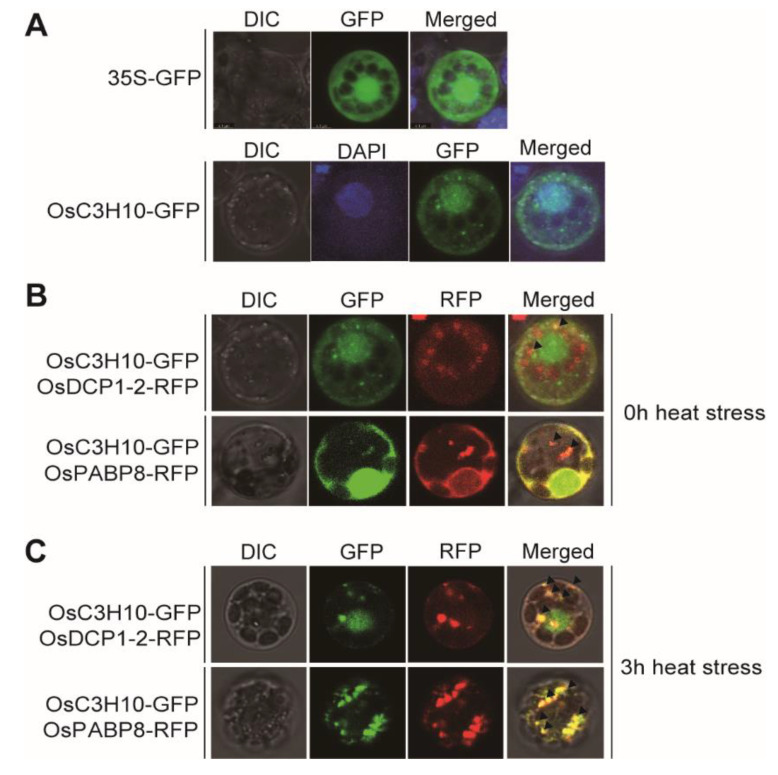
Subcellular localization of OsC3H10 in rice protoplasts. (**A**) Protoplasts were transiently transfected with the *OsC3H10-GFP* expression construct. Transfected protoplasts were stained with DAPI to visualize nuclei. (**B**) The *OsC3H10-GFP* expression construct was cotransformed with *OsDCP1-2-RFP* or *OsPABP8-RFP* into rice protoplasts. (**C**) The transformed protoplasts were incubated at 42 °C for 3 h. The fluorescence of GFP, RFP, DAPI, and chloroplasts was observed in transformed protoplasts using a confocal microscope. Scale bar= 10 μm.

To determine the subcellular localization of OsC3H10, we have generated a construct to express the OsC3H10-GFP translational fusion protein under the control of the *pCaMV 35S* promoter (*OsC3H10-GFP*). The empty vector *p35S-GFP* was used as a negative control to check whether the vector system was working (Figure 2A). The *OsC3H10-GFP* construct was transfected into rice protoplasts. The transformed protoplasts were stained with DAPI to visualize the position of the nucleus. The GFP fluorescence signal of OsC3H10-GFP overlapped with DAPI fluorescence, confirming the nuclear localization of OsC3H10-GFP (Figure 2A). In addition, the GFP fluorescence of OsC3H10-GFP was distributed throughout the cytoplasm and occasionally detected in cytoplasmic foci. To examine whether the association of OsC3H10 with cytoplasmic foci is related to the stress response, we treated protoplasts with heat stress and observed the changes in the OsC3H10 localization patterns (Figure 2A). The stress treatments increased the number of cytoplasmic foci associated with OsC3H10-GFP, while the nuclear localization of OsC3H10-GFP remained, and the density of OsC3H10-GFP in the cytoplasm decreased in the rice protoplasts (Figure 2C). Since the localization of OsC3H10 in the cytoplasmic foci was enhanced by the stress treatments, we hypothesized that OsC3H10 is transported into processing bodies (P-bodies) or stress granules (SGs), both of which are cytoplasmic aggregates involved in stress-mediated mRNA metabolism [19]. To determine whether OsC3H10 is associated with either P-bodies or stress granules, we generated constructs expressing a rice ortholog of Arabidopsis DECAPPING 1 (DCP1) (OsDCP1-2) fused to RFP (*OsDCP1-2-RFP*) to visualize the P-bodies and a rice ortholog of Arabidopsis POLY (A) BINDING PROTEIN 8 (PABP8) (OsPABP8) fused to RFP (*OsPABP8-RFP*) to visualize the stress granules. The *OsC3H10-GFP* construct was cotransformed into the rice protoplasts together with the *OsDCP1-2-RFP* or *OsPABP8-RFP* constructs. RFP fluorescence from both OsDCP1-2-RFP and OsPABP8-RFP were detected in cytoplasmic foci, which are the typical pattern of P-bodies and stress granules; this indicated that the constructs function correctly and can be used as markers for P-bodies and stress granules (Figure 2B,C). The GFP fluorescence of OsC3H10-GFP clearly overlapped with the RFP fluorescence of the P-body marker (OsDCP1-2-RFP) in rice protoplasts after heat stress treatments (Figure 2C). Similarly, OsC3H10-GFP was colocalized with OsPABP8-RFP in rice protoplasts. These results indicate that the localization of OsC3H10 changed from the nucleus/cytoplasm to the cytoplasmic foci in response to stress treatments.

### 2.3. Overexpression of OsC3H10 Enhances Drought Tolerance in Rice

**Figure 3:**
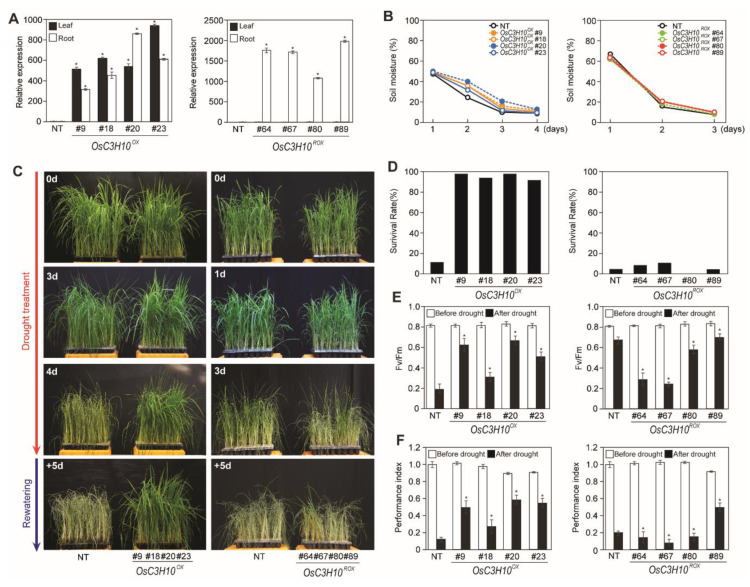
*OsC3H10* overexpression in rice enhances drought tolerance. (**A**) Relative expression levels of *OsC3H10* in nontransgenic (NT) and four independent homozygous T_4_ lines of *PGD1::OsC3H10* (*OsC3H10^OX^*) and *RCc3::OsC3H10* (*OsC3H10^ROX^*) plants. Total RNA extracted from two-week-old rice seedlings was used for qRT-PCR analysis. *OsUbi1* was used as an internal control for normalization. Data represent the mean value ± SD (*n* = 3). (**B**) Measurements of soil moisture content. Data represent the mean value ± SD of thirty measurements performed at different positions in the soil in pots. (**C**) The visual phenotype of the transgenic rice plants during drought treatment. Two-month-old NT plants and transgenic plants from four independent homozygous T_4_ lines of *OsC3H10^OX^* and *OsC3H10^ROX^* were exposed to drought stress for three to four days, followed by rewatering. Numbers on the image indicate the duration of the drought treatment and rewatering. (**D**) The survival rate of the transgenic plants measured 5 days after rewatering. Data represent the mean value ± SD (*n* = 30). (**E**,**F**) Fv/Fm and performance index values of plants under drought conditions. Two-month-old NT plants and transgenic plants from four independent homozygous T_4_ lines of *OsC3H10^OX^* and *OsC3H10^ROX^* were exposed to drought stress for 7 to 9 days (Figure S3). Chlorophyll fluorescence was measured in the dark at the indicated time points after drought treatments using a Handy-plant efficiency analyzer (PEA) fluorometer. Data represent the mean value ± SD (*n* = 30). Significant differences from the NT control are indicated by asterisks (Student’s *t*-test, ^*^p <0.05).

To investigate the physiological functions of *OsC3H10* in response to drought stress, we generated two different types of transgenic rice plants: one with the whole-body overexpression promoter (*OsC3H10^OX^*) and the second with the root-specific overexpression promoter (*OsC3H10^ROX^*). From fifty individual lines of *OsC3H10^OX^* and *OsC3H10^ROX^*, we selected single-copy homozygous T_2_ transgenic lines. Finally, four single-copy homozygous lines of *OsC3H10^OX^* (#9, 18, 20, and 23) and *OsC3H10^ROX^* (#64, 67, 80, and 89) were selected for further study. The expression levels of *OsC3H10* were significantly elevated in the leaves and roots of the *OsC3H10^OX^* transgenic plants, while *OsC3H10* expression increased only in the roots of *OsC3H10^ROX^* transgenic plants, indicating that the transgenic plants were successfully generated as designed (Figure 3A). To test the performance of the selected transgenic plants under drought conditions, two-month-old nontransgenic (NT) and *OsC3H10^OX^* and *OsC3H10^ROX^* transgenic plants were subjected to drought stress by withholding water for three (*OsC3H10^ROX^*) or four days (*OsC3H10^OX^*), followed by rewatering for five days. The soil moisture content showed a similar rate of decrease during the drought treatment, indicating that the stress was uniformly applied to the tested plants (Figure 3B). Under these conditions, drought-induced visual symptoms such as chlorosis, wilting and leaf rolling appeared earlier in NT plants than in *OsC3H10^OX^* (Figure 3C). The *OsC3H10^OX^* transgenic plants showed better recovery from drought-induced damage than NT plants. After 5 days of rewatering, *OsC3H10^OX^* transgenic plants showed an 85 to 90% survival rate, whereas NT plants showed a 14% survival rate (Figure 3D). Since drought stress negatively affects the photosynthetic efficiency of plants [24], we separated each line into different pots and grew the plants until they were 2 months old (Figure S3). We then determined the *Fv/Fm* and the performance index, two different indicators of the photochemical efficiency of the photosystem (Figure 3E,F). *OsC3H10^OX^* plants remained more viable, with higher *Fv/Fm* and performance index values after the drought treatment, than NT plants. In contrast, *OsC3H10^ROX^* transgenic plants showed similar survival rate, Fv/Fm and performance index values as the NT control plants (Figure 3D–F). The same result was shown with agronomic traits as only *OsC3H10^OX^* plants had a significant increase in filling rate (Figure S4). These data suggest that *OsC3H10* overexpression confers drought tolerance in rice plants and that root-specific overexpression of *OsC3H10* was insufficient to confer drought tolerance. In addition, we have tested *OsC3H10^OX^* and *OsC3H10^ROX^* plants for any tolerance to high salinity and ABA. The transgenic plants showed no difference in growth as compared to NT plants under high salinity and ABA conditions (Figure S5).

### 2.4. Identification of Genes Involved in the OsC3H10-Mediated Drought Tolerance Pathway

**Figure 4:**
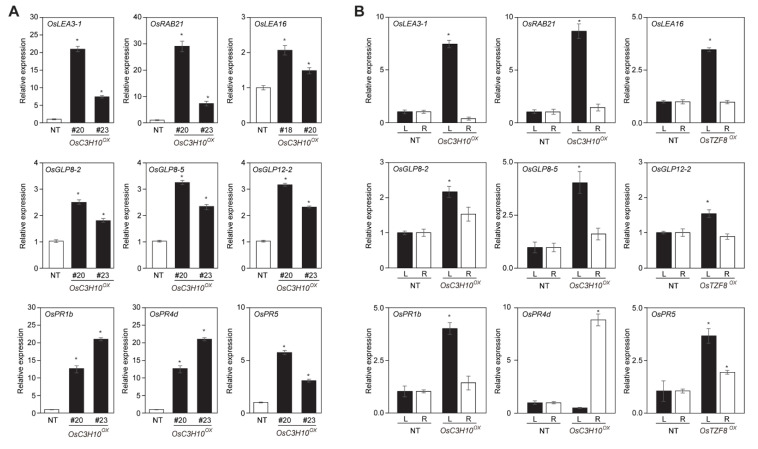
Validation of *OsC3H10*-mediated drought regulatory genes. (**A**) Three-week-old nontransgenic (NT) control and *PGD1::OsC3H10* (*OsC3H10^OX^*) transgenic plants were used for gene expression analysis. (**B**) Leaves and roots of three-week-old nontransgenic (NT) control and *PGD1::OsC3H10* (*OsC3H10^OX^*) transgenic plants were separately harvested for RNA extraction. Rice *UBIQUITIN1* (*OsUBI1*) was used as an internal control for normalization. Data represent the mean value ± standard deviation (SD) (*n* = 3). Significant differences from the nontreated control are indicated by asterisks (Student’s *t*-test, ^*^p < 0.05).

To identify the molecular pathway by which OsC3H10 regulates drought tolerance, RNA sequencing was performed with three-week-old NT control and OsC3H10^OX^ transgenic plants. A cutoff change of 2-fold in expression levels was used to reliably identify the genes regulated by OsC3H10 overexpression. We identified 684 up- and 681 downregulated genes in the OsC3H10^OX^ transgenic plants (Table S2). Since OsC3H10 expression was strongly induced by drought treatment (Figure 1A) and its overexpressors exhibited tolerance to drought stress (Figure 3), we attempted to identify candidate genes that are coregulated by both OsC3H10 overexpression and drought stress. Through comparison of the RNA sequencing profiles of OsC3H10^OX^ transgenic plants with those of NT plants subjected to drought stress for 3 days, sequentially [22] to figure out genes regulated by drought, we identified 179 candidate genes that are positively regulated by both OsC3H10 overexpression and drought stress (Table S3). Of the 681 genes downregulated in OsC3H10^OX^ transgenic plants, 89 genes were downregulated by drought stress (Table S4). The genes upregulated by both OsC3H10 overexpression and drought stress were mainly categorized into three groups: LATE EMBRYOGENESIS ABUNDANT PROTEIN (LEA), GERMIN-LIKE PROTEIN (GLP) and PATHOGENESIS RELATED (PR) genes (Table 1). We also found that additional members of those three groups were upregulated by OsC3H10 overexpression but not by drought treatments. The genes downregulated in the OsC3H10^OX^ transgenic plants were involved in the regulation of transcription and protein phosphorylation as well as in carbohydrate metabolism (Table S4). We examined the expression patterns of several genes selected from the list in OsC3H10^OX^ transgenic plants by independent qRT-PCR analysis. The analysis found their expression patterns to be similar to the expression data derived from RNA sequencing analysis (Figure 4A). We then analyzed the expression levels of these genes in the leaves and roots of NT and OsC3H10^OX^ transgenic rice plants (Figure 4B). Interestingly, the upregulation of the tested genes, except OsPR4d, was more obvious in the leaves than in the roots of the OsC3H10^OX^ transgenic plants. These results suggest that OsC3H10 modulates the expression of drought-related genes predominantly in leaves, thereby contributing to enhanced drought tolerance.

## 3. Discussion

CCCH ZF proteins are known to be involved in the regulation of growth, development and stress responses in plants. In Arabidopsis, the functions of several CCCHZF members has been characterized [7]. For example, *AtTZF1* affects ABA- and gibberellin-mediated growth and stress responses [8,25]. *AtTZF4, 5* and *6* are involved in the regulation of seed germination [9]. In rice, in contrast, *OsTZF1* is the only gene that is functionally characterized as a regulator of the stress response [10]. Thus, further characterization of *OsCCCHZFs* is crucial for understanding the molecular mechanisms governed by *TZF*s in rice. In this study, we identified *OsC3H10* as a drought-induced *TZF* gene. *OsC3H10* and *OsC3H52* are distinguished from other annotated *OsTZF*s due to the variations in their TZF motifs and their different developmental expression patterns [7]. Among nine annotated *OsCCCHZF*s, *OsC3H10* was found to show the most sensitive response to drought stress (Figure 1A, Table S1). The expression of *OsC3H10* was also induced by high salinity and ABA (Figure 1B,C), suggesting that *OsC3H10* could participate in multiple stress responses. Interestingly, *OsC3H10* was more induced in leaves than roots by the drought stress and higher in roots by the ABA treatments, respectively (Figure 1B,D). *OsC3H37* expression was similarly induced in leaves and roots by the drought stress and ABA treatments, respectively (Figure S1A,B). The *OsC3H10* promoter contains three DREs and two ABREs (Figure S1C). DREs are well-known *cis*-elements that are regulated by ABA-independent drought-induced *OsDREB2* transcription factors [26,27]. Thus, the drought-induced expression of *OsC3H10* could be regulated in an ABA-independent manner.

The functions of CCCHZF proteins are closely related to their subcellular localization [10,18]. For example, mammalian CCCHZFs are nucleocytoplasmic shuttling proteins that can localize to the nucleus and cytoplasmic speckle structures, such as processing bodies (PBs) and stress granules (SGs) [20,28]. PBs and SGs are cytosolic aggregations of messenger ribonucleoprotein complexes (mRNPs) that play crucial roles in RNA metabolism under stress conditions [19,29]. In plants, Arabidopsis AtTZF1, AtTZF4, AtTZF5, and AtC3H50 and rice OsTZF1/OsC3H35 showed localization in PBs and SGs [9,10,18]. Here, we also found that OsC3H10 is colocalized with OsDCP1-2 and OsPABP8, which are markers for PBs and SGs, respectively (Figure 2). OsC3H10 was predominantly localized in the nucleus under normal conditions and was transported into cytoplasmic foci under stress conditions. These data indicate that the expression and the subcellular localization of OsC3H10 are dynamically regulated by environmental conditions.

To determine the function of *OsC3H10* in response to drought, we investigated the performance of transgenic plants overexpressing *OsC3H10* under drought conditions (Figure 3). We compared the effects of tissue-specific overexpression of *OsC3H10* on drought tolerance by generating transgenic plants that overexpress *OsC3H10* either throughout the entire plant (*OsC3H10^OX^*) or specifically in the roots (*OsC3H10^ROX^*) (Figure 3A). The results demonstrated that the whole-body overexpression of *OsC3H10* enhanced drought tolerance in rice, but the root-specific overexpression of *OsC3H10* was insufficient to confer drought tolerance in rice (Figure 3C–F). These observations led us to speculate either that the overexpression of *OsC3H10* in shoots is sufficient to confer drought tolerance or that the overexpression of *OsC3H10* in shoots and roots additively improves drought tolerance. We found that the overexpression of *OsC3H10* in leaves is more efficient at inducing the expression of downstream genes involved in the *OsC3H10*-mediated drought tolerance pathway (Figure 4B), confirming that the overexpression of *OsC3H10* in shoots is crucial for conferring drought tolerance. These observations also suggest that overexpression of *OsC3H10* in roots is insufficient to activate the *OsC3H10*-mediated drought tolerance pathway, as most of the expression of the target genes showed no difference between NT and ROX lines in both leaves and roots (Figure S6). We found that *OsC3H10* expression was predominantly induced in leaves by drought treatments (Figure 1A). It has been reported that coregulation of the TZF protein and its interacting protein is required for stress responses [30,31]. For example, AtTZF5 interacts with the mediator of ABA-regulated Dormancy1 (MARD1) and Responsive to Dehydration 21A (RD21A), both of which are coregulated with *AtTZF5* by developmental and stress signals [31]. In addition, the phosphorylation of AtTZF9 by a mitogen-activated protein kinase is required to trigger a full PAMP-triggered immune response [14]. Further biochemical analysis (e.g., identification of interacting proteins) will provide clues to the tissue-specific regulation of *OsC3H10*-mediated drought tolerance.

The transcriptome analysis of the *OsC3H10^OX^* plants revealed the upregulation of many stress-related genes in the transgenic plants (Table 1, Table S2, Table S3 and Figure 4A). The downstream genes could be divided into three groups: LATE EMBRYOGENESIS ABUNDANT PROTEIN (LEA), PATHOGENESIS RELATED (PR) and GERMIN-LIKE PROTEIN (GLP) genes. The list included seven *LEA* genes, which are representative drought-induced genes that can enhance drought tolerance when overexpressed by acting as hydrophilins that provide water-holding capacity and subcellular matrix protection under stress conditions [32,33,34,35,36]. In addition to the upregulation of *LEA* genes, the expression of eleven *PR* genes was upregulated in *OsC3H10^OX^* plants. *PR* genes are the main components of plant defense mechanisms against biotic stresses, but their functions in abiotic stress have also been reported in plants [37,38,39]. Specifically, the overexpression of Arabidopsis *PR1*, *PR2* and *PR5* in *Arabidopsis* enhances drought tolerance [37]. Similarly, the overexpression of two rice *OsPR10* family genes conferred drought tolerance in rice plants [38,40]. Moreover, a mutation in *NONEXPRESSER OF PR GENES 1* (*NPR1*), an upstream regulator of the *PR* gene, reduced drought tolerance in tomato [39]. Thus, the upregulation of *LEA* and *PR* genes by *OsC3H10* overexpression could contribute to enhanced drought tolerance. *GERMIN LIKE PROTEIN* (*GLP*) genes were also upregulated in the *OsC3H10^OX^* plants (Table 1 and Figure 4A). GLP was initially identified as a marker of germination in wheat embryos [41] but has been further characterized as a glycoprotein with oxalate oxidase and superoxide dismutase activity (SOD) [42,43]. It has been proposed that *GLP*s participate in the defense response against pathogen invasions in plants by regulating ROS levels through SOD activity [44]. Although the molecular functions of *GLP*s in the drought tolerance pathway are still not fully understood, there are several lines of evidence proposing the involvement of *GLP*s in abiotic stress responses. Transcriptome analysis revealed that several *AtGLP*s and *OsGLP*s contain stress-responsive elements on their promoters, and their expression is regulated by various abiotic stresses [43,45]. In addition, the overexpression of peanut *AhGLP*s and soybean *GmGLP7* enhanced abiotic stress tolerance in Arabidopsis [46,47]. Thus, the upregulation of *OsGLP*s by the overexpression of *OsC3H10* might be involved in drought tolerance mechanisms. We cannot rule out the possibility that the upregulation of *OsGLP*s in *OsC3H10^OX^* plants is related to germination regulation in rice plants, since *OsC3H10* expression was dynamically regulated during the germination process (Figure 1E). However, as the seed germination percentage showed no difference from that of the nontransgenic line and *OsC3H10^OX^* plants seemed to germinate slightly slower than NT plants, *OsC3H10* may be related to storage functions. Further characterization of *GLP*s will be required to determine the functions of *GLP*s in the *OsC3H10*-mediated drought tolerance pathway.

In addition to *OsTZF1*, *OsC3H10, OsC3H37*, and *OsC3H50* also showed drought-induced expression patterns, indicating that several *OsTZF*s are implicated in the drought response in rice. It has been reported that *OsTZF1* regulates drought tolerance in rice by regulating the expression of stress-related genes [10]. Interestingly, there is a clear overlap between the *OsTZF1* and *OsC3H10* mediated drought tolerance pathways. The majority of stress-related genes upregulated by *OsTZF1* were also upregulated in *OsC3H10^OX^* plants (Table S5). The list included *LEA* and *GLP* genes. In addition, three genes related to ROS production were also coregulated by *OsTZF1* and *OsC3H10* (Table S5). Moreover, several *PR* genes were also identified as downstream genes of *OsTZF1* [10]. On the other hand, no clear overlap was found in other categories regulated by *OsTZF1* and *OsC3H10* other than in the category of stress-related genes. These results suggest that *OsTZF1* and *OsC3H10* share downstream pathways that are involved in drought tolerance but may play distinct roles in other processes.

## 4. Materials and Methods

### 4.1. Plant Materials and Plasmid Construction for Rice Transformation

*Oryza sativa* cvs. Nipponbare and Ilmi were used as the plant materials in this study. The rice seeds (*Oryza sativa* cvs Nipponbare and Ilmi) were purchased from the Rice Genome Resource Center, Japan. *Oryza sativa* cv Ilmi seeds were originally obtained from the Rural Development Administration, Korea. To generate *OsC3H10*-overexpressing transgenic rice plants, the *OsC3H10* (Os01g0738400) coding sequence was amplified from rice (*Oryza sativa* cv Nipponbare) cDNA using PrimeSTAR (Takara, Japan). The amplified *OsC3H10* fragment was cloned into the *pSB11-PGD1* vector for whole-body overexpression and into the *pSB11-RCc3* vector for root-specific overexpression [48]. Transgenic rice plants were generated by the Agrobacterium-mediated cocultivation method as described previously [49]. Copy numbers of the transgenic plants were determined by TaqMan Q-PCR (Thermo Fisher) using probes specific for the *bar* gene as previously described [50] (Figure S7). The selected single-copy insertion lines were self-fertilized, and homozygous transgenic lines were selected from the T_2_ generations by examining segregation rates on MS media containing phosphinothricin (Duchefa) (*n* = 20). Four independent single-copy inserted homozygous plants were selected and propagated in a rice paddy field at Kyungpook National University, Gunwi (128:34E/36:15N), Korea, each line checked with proper vector using genomic PCR (Figure S6). Information about the primers used for plasmid construction is listed in Table S6.

### 4.2. Plant Growth and Abiotic Stress Treatments

Rice seeds (*n* = 10) (*Oryza sativa* cv Ilmi) were planted on Murashige–Skoog (MS) solid medium and incubated in the dark for 3 days at 28 °C. After germinating at 28 °C, seedlings were transplanted into soil pots (4 × 4 × 6 cm; two plants per pot) and grown for two weeks in a green house, set with long day conditions (16h light, 8h dark). For the abiotic stress treatments, the soil was removed from the roots of all 10 seedlings with its order randomized to minimize biases. Drought stress was induced by air-drying the seedlings; the salinity stress and ABA treatments were imposed by incubating the seedlings in water containing 400 mM NaCl (Sigma, USA) or 100 μM ABA (Duchefa, Netherlands), respectively, at 28 °C. Low-temperature stress was induced by incubating the seedlings in a 4 °C incubator. The seedlings were harvested at the indicated time points after the abiotic stress treatments and were immediately frozen in liquid nitrogen for RNA extraction.

### 4.3. Evaluation of Drought Tolerance in Rice Plants

*OsC3H10^OX^* and *OsC3H10^ROX^* transgenic and NT control plants (*Oryza sativa* cv. Nakdong for *OsC3H10^OX^*; *Oryza sativa* cv. Ilmi for *OsC3H10^ROX^*) were germinated on MS media at 28 °C for 3 days. Thirty plants from each line were transplanted into ten soil pots (4 × 4 × 6 cm; three plants per pot) within a container (59 × 38.5 × 15 cm) and grown for two months in a greenhouse at 28–30 °C. Drought stress was imposed by removing the pots from the container for the indicated periods and rewatering for 5 days [51]. Drought-induced symptoms were visualized using a NEX-5N camera (Sony, Japan), and the soil moisture was measured using a SM150 soil moisture sensor with five repeats of each line in a random spot of each pot (Delta T Devices, UK) at the indicated time points.

### 4.4. JIP Analysis

To measure the chlorophyll a fluorescence and the performance index, 3 of the 3-week-old plants were moved from small pots to large pots and grown until they were 2 months old. The longest leaves from each plant at their apex, middle and base regions were analyzed using a Handy-PEA fluorimeter (Hansatech Instrument, UK). After dark-adaption of the plant for at least 1 h to provide a sufficient opening time for the RCs to be fully oxidized, twenty readings per line were averaged using Handy-PEA software (version 1.31). Chlorophyll a fluorescence (Fv/Fm) and the performance index were calculated according to the equations of the JIP test [52]. The performance index was normalized to the NT value before drought. Each line was analyzed every day, with 20 reads in randomly chosen different leaves of each line. Each measurement continued for 8 days for OsC3H10^OX^ and 6 days for OsC3H10^ROX^.

### 4.5. Quantitative Real-Time PCR Analysis

The total RNA was extracted from rice plants harvested at the indicated time points using TRIzol reagent (Invitrogen, USA) according to the manufacturer’s instructions. To generate first-strand complementary DNA (cDNA), 1 μg of total RNA was reverse-transcribed using first-strand cDNA RevertAid M-MuLV reverse transcriptase (Thermo Scientific, USA). Quantitative real-time PCR (qRT-PCR) was performed with 2X Real-Time PCR smart mix (SolGent, Korea) and 20X EvaGreen (SolGent, Korea). The PCRs were performed by initial denaturation at 95 °C for 10 min, followed by forty cycles of 95 °C for 20 s, 60 °C for 20 s and 72 °C for 30 s using a Mx300p real-time PCR system (Stratagene, USA) and Mx3000p software v2.02 (Stratagene, La Jolla, CA). Rice *UBIQUITIN1* (Os06g0681400) was used as an internal control for normalization. Each sample was triplicated and averaged. After PCR, data were comparatively quantified and calibrated using Mx3000p software v2.02 (Stratagene, La Jolla, CA). Each gene expression used the 2^−ΔΔCT^ method of Livak and Schmittgen (2001) [44], based on the threshold cycle (CT) which is the cycle of the fluorescence level when it reaches a certain amount (the threshold). A housekeeping gene, in this paper ubiquitin, which is expressed similarly in all the tissue, is used as a reference gene to normalize as a guideline to prevent differences in expression levels due to the quantity of DNA/RNA and to reduce variation caused by PCR processes. By deleting the CT value of ubiquitin of each sample from the CT value of C3H10 gene (ΔCT), the exact Ct value of each sample remains to be compared. The ΔCT value of 10 day leaf was highest among other tissues indicating its expression level is the lowest, which was the reason for considering its level of expression to be 1 to compare the pattern of different tissues. The differences between the CT value of 10-day leaf and other CT values of other tissues (Δ ΔCT) were converted to 2−Δ ΔCT value to see the exact difference in expression levels. Each condition was done with two biological repeats using the same cDNA. Information about the primers used for qRT-PCR analysis is listed in Table S6.

### 4.6. Rice Protoplast Isolation and Transient Gene Expression

Rice seedlings (*Oryza sativa* cv. Ilmi), 20 seedlings planted in 10 different plates, were grown in the dark for 10 days and transferred to light conditions for 10 h. Rice protoplast preparation and transient gene expression were performed as described previously [51]. For transient expression of *OsC3H10-GFP* in rice protoplasts, the *OsC3H10* coding sequence translationally fused with GFP (OsC3H10-GFP) was inserted into the *pHBT* vector carrying the 35S promoter [51,52,53,54]. Similarly, coding sequences of *OsDCP1-2* and *OsPABP8* were translationally fused with red fluorescent protein (RFP) and inserted into the *pHBT* vector. Information about the primers used for plasmid construction is listed in Table S6. The constructs were transformed into protoplasts using polyethylene glycol (PEG)-mediated transformation [55,56]. The transformed protoplasts were treated with heat stress by incubation at 42 °C for 3 h. The subcellular localization of OsC3H10 was monitored by using a Leica SP8 stimulated emission depletion (STED) laser scanning confocal microscope (Leica, Germany).

### 4.7. RNA Sequencing Analysis

The total RNA was extracted from whole *OsC3H10^OX^* (T_4_ generation, line number #20) and nontransgenic (NT cv. Nakdong) plants using an RNeasy plant mini kit (Qiagen, Germany) according to the manufacturer’s instructions. cDNA library preparation and RNA sequencing analysis were performed as previously described [51]. cDNA libraries were prepared from the total RNA using the TruSeq RNA sample prep kit (v2) (Macrogen, Korea). Two biological replicates were analyzed by RNA sequencing analysis. Single-end sequences were obtained using IRGSP (v 1.0), and raw sequence reads were trimmed to remove adaptor sequences; sequences with a quality lower than Q20 were removed using Trimmomatic 0.32 software [53]. To map the reads to the reference genome, all reads were assembled with annotated genes from the Rap-DB database (http://rapdb.dna.affrc.go.jp; IRGSP (v 1.0)) using TopHat software (https://ccb.jhu.edu/software/tophat/index.shtml). After mapping the reads to a reference genome, differentially expressed genes (DEGs) were selected using two conditions: a cut-off change of at least 2-fold between the NT and transgenic lines and an independent T-test *p*-value < 0.05. The selected DEGs were grouped by hierarchical clustering analysis (complete linkage). The data set can be obtained from the GEO database with series accession number GSE135940 (http://www.ncbi.nlm.nih.gov/geo/).

## 5. Conclusions

In this study, we suggested that *OsC3H10* controls drought tolerance by modulating the expression of stress-related genes involved in multiple drought-tolerant pathways. The genes include *LATE EMBRYOGENESIS ABUNDANT PROTEINs* (*LEAs*), *PATHOGENESIS RELATED GENEs* (*PRs*) and *GERMIN-LIKE PROTEINs* (*GLPs*), all of which have been well characterized as important genes mediating drought tolerance responses in plants. We also found that the root-specific overexpression of *OsC3H10* is insufficient to induce drought tolerance, while the overexpression of *OsC3H10* throughout the entire plant improves drought tolerance in plants. Moreover, the overexpression of *OsC3H10* in leaves is more efficient than that in the roots in inducing the expression of downstream genes involved in the *OsC3H10*-mediated drought tolerance pathway. All the results indicated that the *OsC3H10* gene plays an important role in the regulation of genes involved in drought tolerance in rice.

## Figures and Tables

**Table 1:** List of genes upregulated (> 2-fold) in *OsC3H10^OX^* transgenic rice in comparison with their expression in nontransgenic plants.

Gene	Description ^a^	Drought_2d ^b^
**LEAs**		
Os05g0542500	OsLEA3-1	Up ^c^
Os11g0454300	OsRAB16A	Up
Os11g0454200	OsRAB16B	Up
Os01g0705200	OsLEA3	Up
Os03g0168100	OsLEA16	Up
Os04g0589800	OsLEA1	Up
Os11g0451700	OsRAB21	Up
**GLPs**		
Os08g0190100	OsGLP8-11	Up
Os12g0154900	OsGLP12-3	ND ^e^
Os12g0154700	OsGLP12-1	ND
Os08g0189700	OSGLP8-8	Up
Os12g0155000	OsGLP12-4	ND
Os08g0189100	OsGLP8-2	Up
Os12g0154800	OsGLP12-2	ND
Os08g0189400	OsGLP8-5	ND
**PRs**		
Os07g0127700	OsPR1b	ND
Os11g0591800	OsPR4d	ND
Os03g0661600	Pathogenesis-related protein class 5 gene	Up
Os12g0628600	OsPR5	Down
Os12g0555000	OsPR10A	Up
Os12g0555200	OsPR10B	DOWN
Os03g0663400	Similar to Thaumatin-like protein	Up
Os07g0127600	OsPR1-73	ND
Os05g0375400	Beta-glucanase precursor.	Up
Os11g0592000	Similar to Barwin.	Up
Os12g0437800	Similar to MPI.	Up

^a^ Functional description of genes based on RAP-DB; ^b^ Drought response of genes determined based on RNA sequencing analysis of rice plants treated with drought for 2 days [22]; ^c^ Upregulated by drought; ^d^ Downregulated by drought; ^e^ Not determined; ^f^ Not changed.

## Data Availability

RNA sequencing data that support the findings of this study have been deposited in the GEO database with accession number GSE135940 (http://www.ncbi.nlm.nih.gov/geo/). The other datasets used and/or analyzed during the current study are available from the corresponding author upon reasonable request.

## Supplementary Materials

The following are available online at https://www.mdpi.com/2223-7747/9/10/1298/s1, Figure S1: Expression patterns of *OsTZF*s in response to drought and ABA. A-B Two-week-old rice seedlings (Oryza sativa L. Japonica cv. Ilmi) were exposed to drought (A) and 100µM abscisic acid(ABA) (B). Leaves and roots of rice plants were harvested at the indicated time points after the treatments for gene expression analysis. Data represent the mean value ± standard deviation (SD) (*n* = 3). (C) Promoter analysis of Os*CCCHZF*s for DRE and ABRE *cis*-elements. Figure S2: Germination rates of NT and overexpression lines. Oryza sativa cv Nakdong and Ilmi were used as the plant material. Rice seeds (*n* = 100) were planted on a Murashige-Skoog (MS) solid medium and incubated in the dark for 4 days at 28℃. The seed was considered germinated if both cotyledon and root tissue had developed. No dramatic differences in germination, only the time interval, between the NT was observed. Figure S3: 2-month-old NT and *OsC3H10* overexpressing rice plants under drought condition. Drought stress was induced in 2-month old NT and OsC3H10 OX lines (A). 1 plant per pot for 8 days and ROX lines, (B) 3 plants per pot for 9 days. Chlorophyll a fluorescence (Fv/Fm) and the performance index were measured once in two days (*n* = 20). Figure S4: Agronomic traits of PGD1:OsC3H10 and RCc3:OsC3H10 plants grown in the field under drought conditions. Each spider plots are the agronomic traits of four independent homozygous T4 lines of PGD1: OsC3H10, T3 lines of RCc3: OsC3H10 plants and corresponding NT controls under drought conditions. Each data point represents the percentage of the mean value (*n* = 10). The mean measurements from the NT controls were assigned a 100% reference value. CL, Culm length; PL, Panicle Length; NP, Number of penicles per hill; Number of Filled Seeds; TNS, Total Number of Spikelets; TSW, Total Seed Weight; FR, Filling Rate; 1000 SW, 1000 Seed Weight. Figure S5: Germination assay of OsC3H10 OX lines and ROX lines under NaCl and ABA treatment. A. The upper panel is the growth size comparative pictures of NT with OsC3H10 OX line and ROX lines grown under 0mM, 100mM, 200mM NaCl treated media for 7 days and lower panel is shoot and root lengths of plants in average (*n* < 50). B. The upper panel is the growth size comparative picture of NT with OsC3H10 OX lines and ROX lines grown under 0µM, 5µM, 10µM ABA treated media for 7 days and lower panel shoot and root lengths of plants in average(*n* < 50). Figure S6: Target validation in the leaves and roots of the ROX line. Two-week-old nontransgenic (NT) control and RCc3:OsC3H10 (OsC3H10^ROX^) transgenic plants were used for target validation. Rice *UBIQUITIN 1(OsUbi1)* was used as an internal control for normalization. Significant differences from the nontreated control are indicated by asterisks (**p* < 0.05). Figure S7: TaqMan PCR results for transgenic lines. Copy number of (A) *OsC3H10^OX^* and (B) *OsC3H10^ROX^* transgenic lines were determined using Taqman PCR by bar probes and genomic DNA of single-copy known homozygotes and hemizygotes as positive controls. Figure S8: Genomic PCR of NT and OsC3H10 transgenic lines. 3 different set of primers were used for molecular confirmation of vectors inserted in each transgenic line. NT was used as negative control to confirm that no sequencial similarity exist in NT. Table S1. indicates expression levels of other members of the OsTZF family after drought stress treatments which was obtained from previous RNA sequencing data [22], presented in Log_2_ ratio normalized by 0 day expression level. Table S2. is a list of genes regulated by *OsC3H10* overexpression. Table S3. is a list of genes upregulated by both *OsC3H10* overexpression and drought stress. Table S4. is a list of genes downregulated by both *OsC3H10* overexpression and drought stress. Table S5. is a list of genes upregulated by both *OsTZF1* and *OsC3H10* overexpression. Table S6 contains information on primers used in this study.

## Abbreviations

TZF	Tandem CCCH zinc finger
ABA	Abscisic acid
DRE	Dehydration-responsive element
ABRE	ABA-responsive element
DAPI	4′,6-diamidino-2-phenylindole
DIC	Differential Interference Contrast
PB	Processing body
SG	Stress granule
DCP1	Decapping 1
PABP8	Poly(A) binding protein 8
NT	Nontransgenic
LEA	Late embryogenesis abundant protein
GLP	Germin-like protein
PR	Pathogenesis related
MPI	Maize protease inhibitor
RAP-DB	Rice annotation project-database
