# Supplementary figures and images for: Overexpression of OsC3H10, a CCCH-Zinc Finger, Improves Drought Tolerance in Rice by Regulating Stress-Related Genes
Source: Plants (Basel). 2020 Oct 1;9(10):1298. doi: 10.3390/plants9101298 (PMC7599559; doi:10.3390/plants9101298)

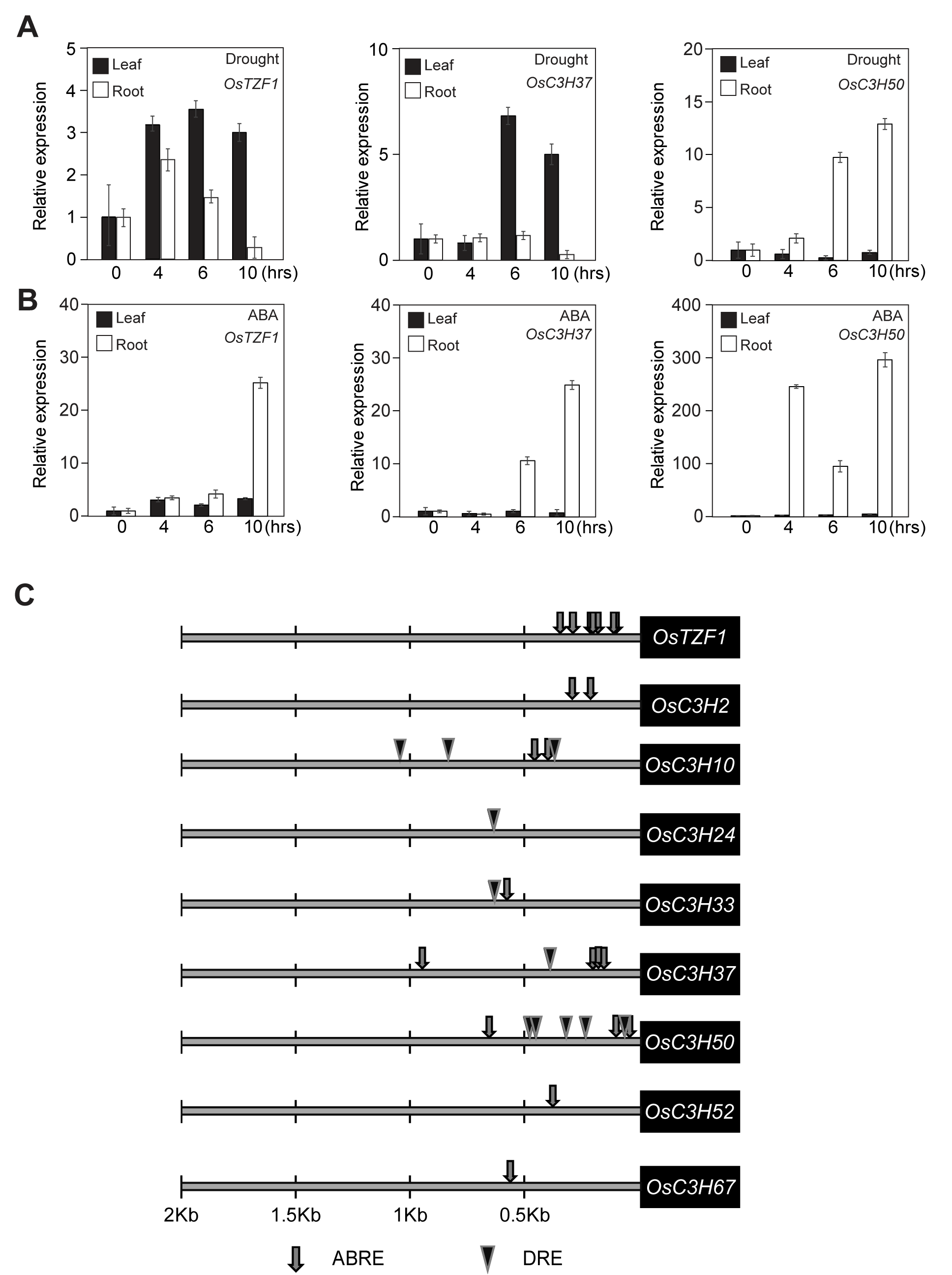

Supplement: Supplementary file 1 [file plants-09-01298-s001.zip › Figure S1.tif]

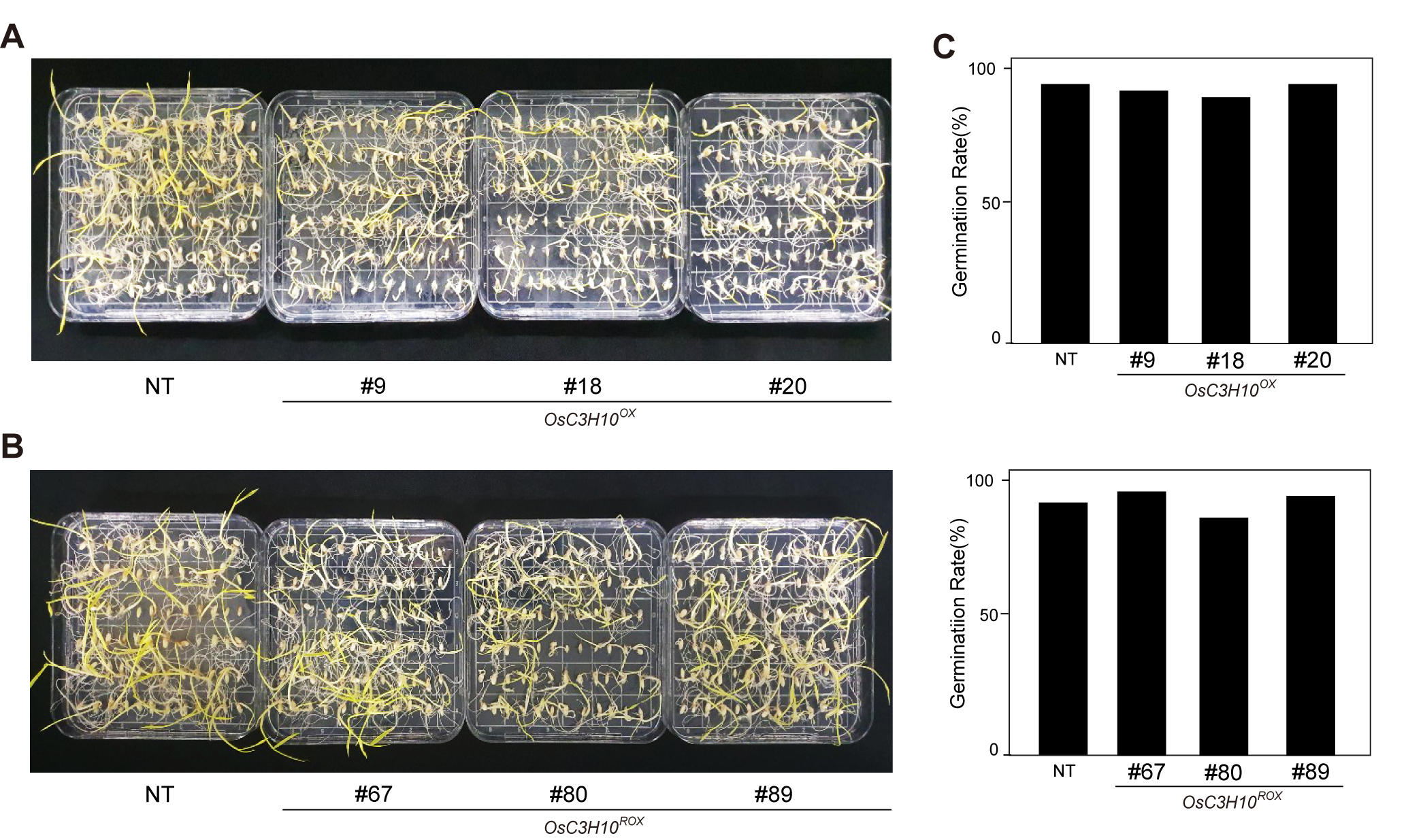

Supplement: Supplementary file 1 [file plants-09-01298-s001.zip › Figure S2.tif]

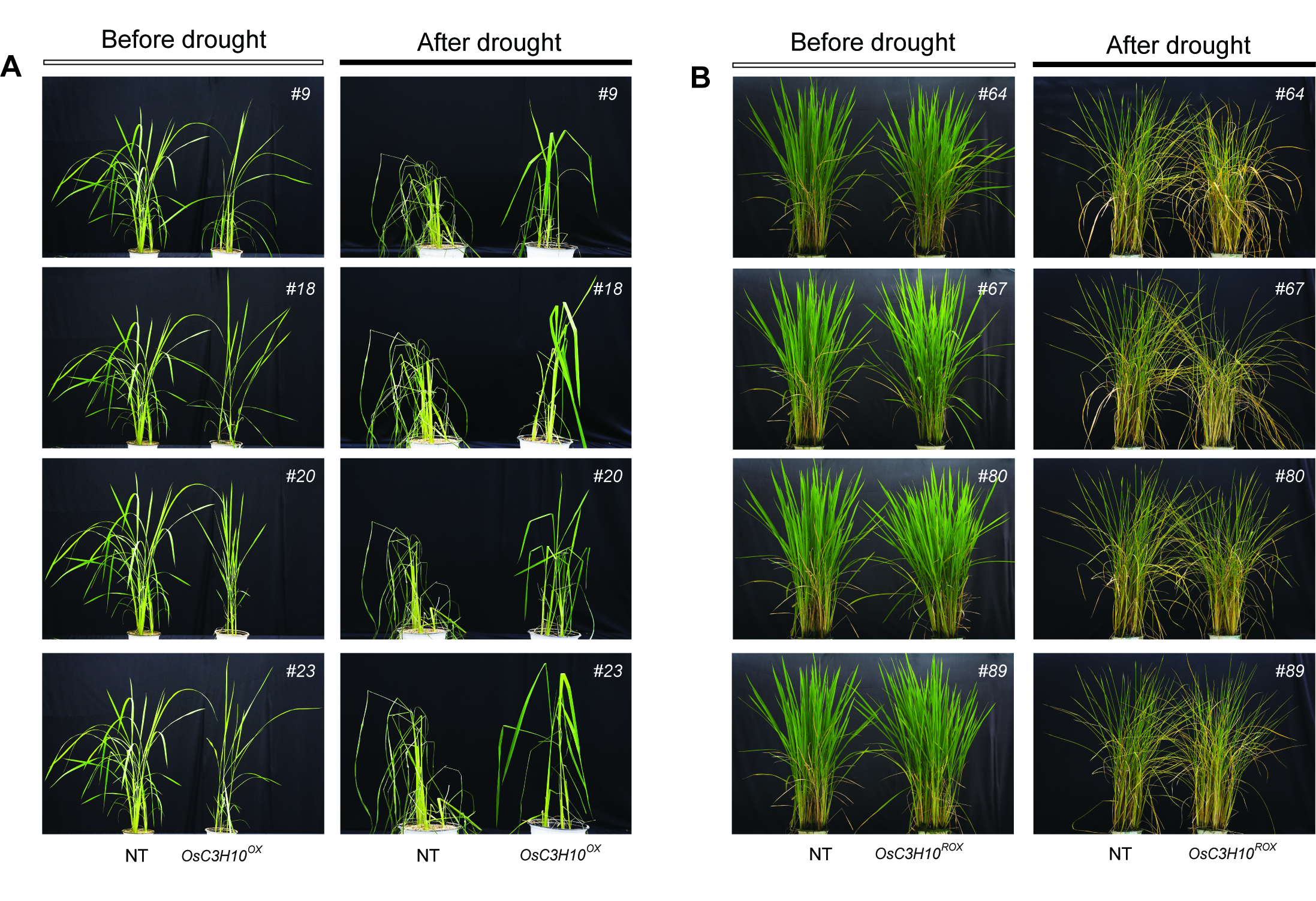

Supplement: Supplementary file 1 [file plants-09-01298-s001.zip › Figure S3.tif]

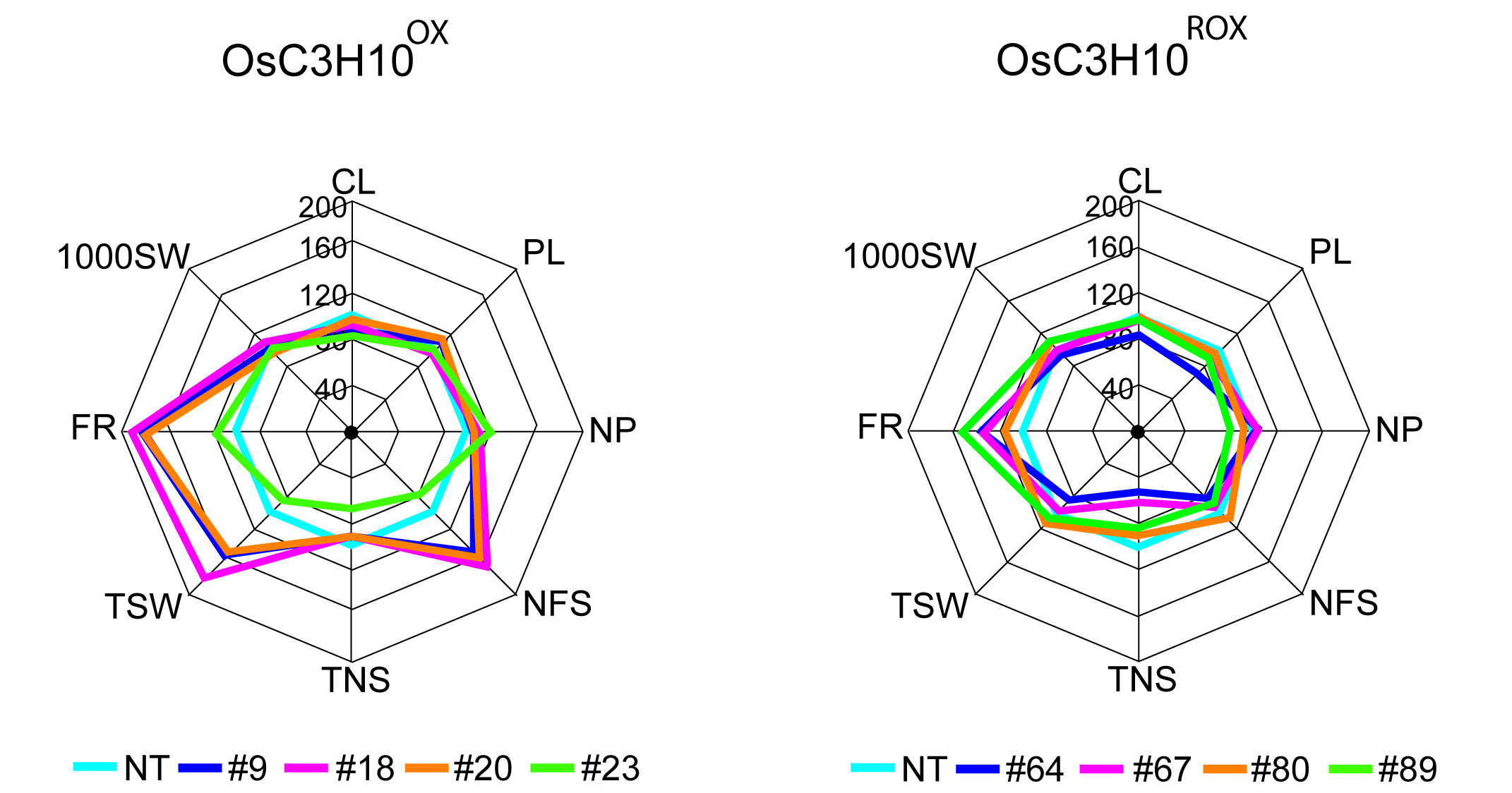

Supplement: Supplementary file 1 [file plants-09-01298-s001.zip › Figure S4.tif]

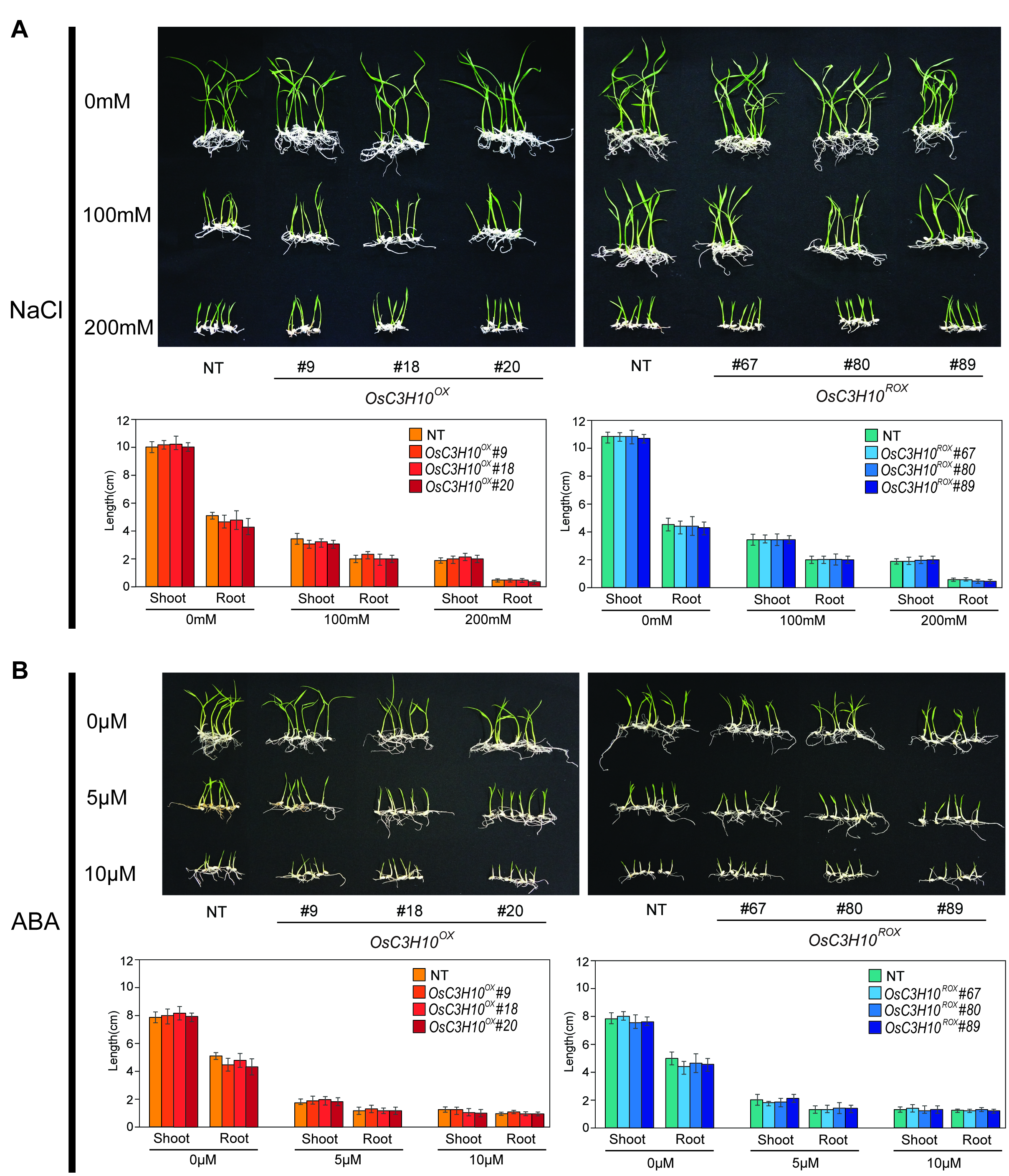

Supplement: Supplementary file 1 [file plants-09-01298-s001.zip › Figure S5.tif]

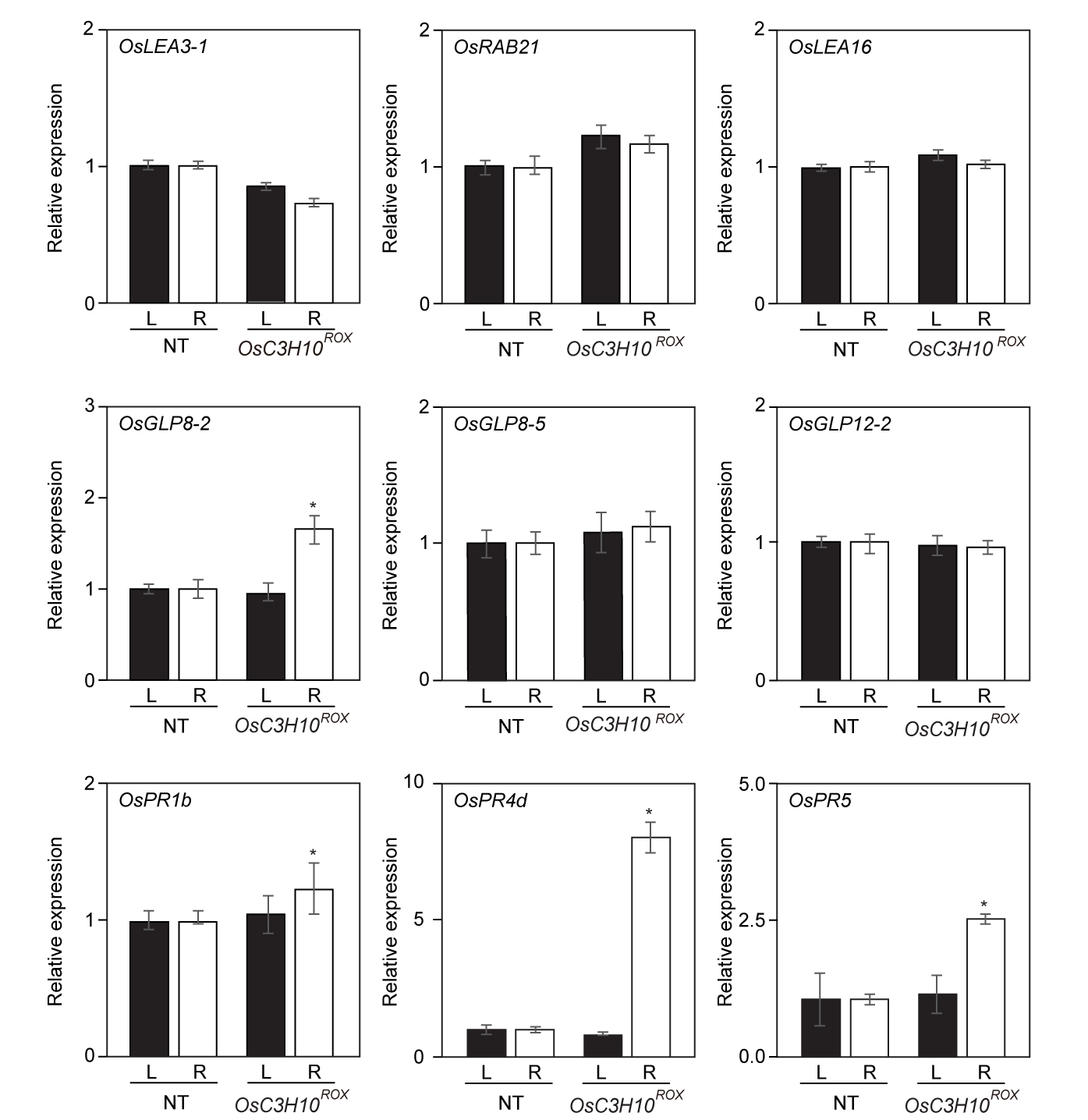

Supplement: Supplementary file 1 [file plants-09-01298-s001.zip › Figure S6.tif]

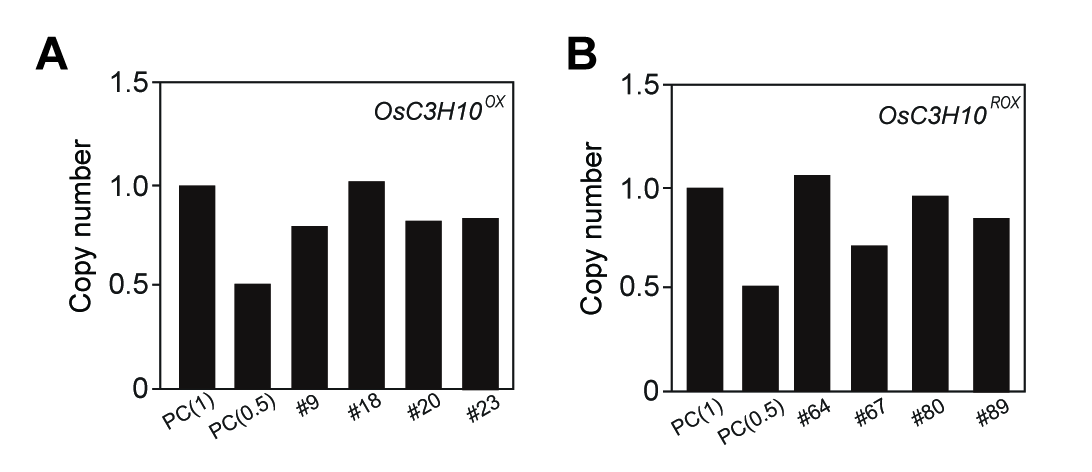

Supplement: Supplementary file 1 [file plants-09-01298-s001.zip › Figure S7.tif]

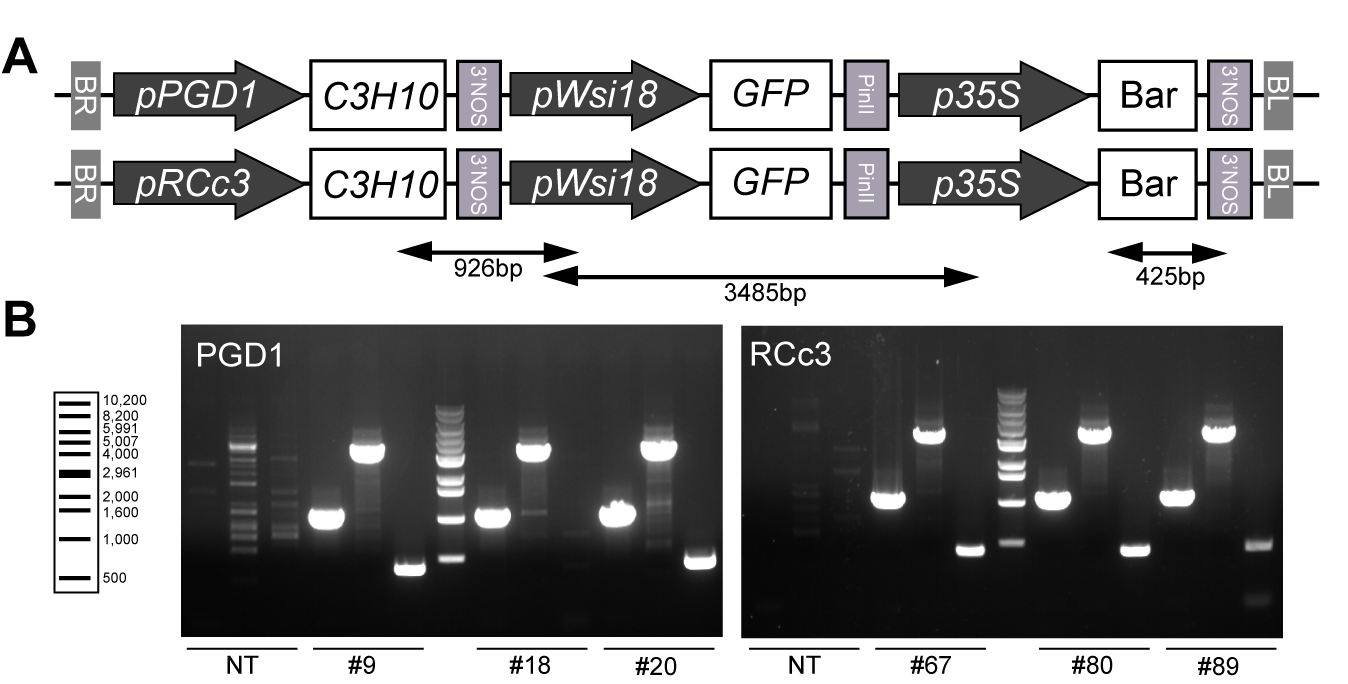

Supplement: Supplementary file 1 [file plants-09-01298-s001.zip › Figure S8.tif]

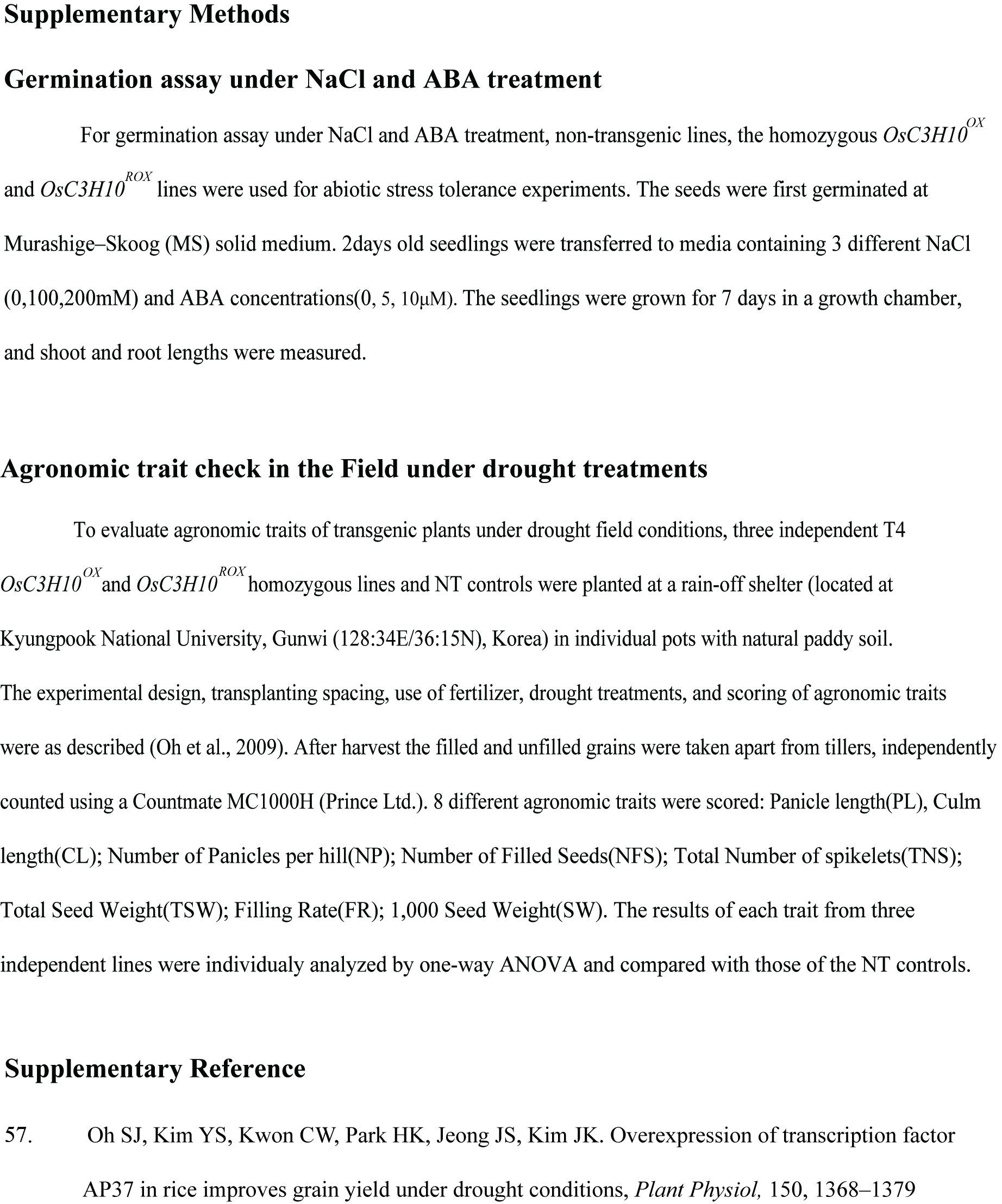

Supplement: Supplementary file 1 [file plants-09-01298-s001.zip › Supplementary Methods.tif]
